# Influence of maternal and own genotype at tanning dependence-related SNPs on sun exposure in childhood

**DOI:** 10.1186/s12881-018-0575-z

**Published:** 2018-04-12

**Authors:** Jasmine Khouja, Sarah J. Lewis, Carolina Bonilla

**Affiliations:** 1 MRC Integrative Epidemiology Unit, University of Bristol, Bristol, UK; 2 Bristol Medical School: Population Health Sciences, University of Bristol, Bristol, UK; 3 School of Experimental Psychology, University of Bristol, Bristol, UK; 4 Departamento de Medicina Preventiva, Faculdade de Medicina, Universidade de São Paulo, São Paulo, Brazil

**Keywords:** Tanning dependenceMaternal, SNPs/single nucleotide polymorphisms, Genotypes, ALSPAC

## Abstract

**Background:**

Research suggests there may be a genetic influence on the likelihood of becoming tanning dependent (TD). The way in which mothers regulate their children’s sun exposure may be affected by being TD. We investigated the associations between single nucleotide polymorphisms (SNPs) related to being TD and early sun exposure.

**Methods:**

Data from the Avon Longitudinal Study of Parents and Children (ALSPAC) were used. Associations between 17 TD related SNPs in children and their mothers and 10 sun exposure variables in children (assessed via questionnaire at age 8) were analyzed in logistic and ordinal logistic regressions. Analyses were adjusted for principal components of population structure and age (at time of questionnaire response). Models with additional adjustment for maternal or offspring genotypes were also tested. Secondary analyses included adjustment for sex and skin pigmentation.

**Results:**

Among ALSPAC children, the rs29132 SNP in the Vesicle-associated membrane protein-associated protein A (*VAPA*) gene was associated with five sun exposure variables whilst the rs650662 SNP in the Opioid Receptor Mu 1 (*OPRM1*) gene was associated with three. The remaining SNPs did not show associations beyond what was expected by chance. After Bonferroni correction one SNP in the children was associated with an increased likelihood of using sun cream whilst in the sun at 8 years old (rs60050811 in the Spermatogenesis and Centriole Associated 1 (*SPATC1*) gene, OR per C allele = 1.34, 95% CI 1.11–1.62, *p* = .003). In the mothers, rs650662 in *OPRM1* was associated with the use of a lower factor of sun cream in their children, (OR per A allele = 0.89, 95% CI 0.82–0.96, *p* = .002). Whilst rs2073478 in the Aldehyde Dehydrogenase 1 Family Member B1 (*ALDH1B1*) gene was associated with a reduced odds of their child using a sun block or cream with a 4 star rating (OR per T allele = 0.68, 95% CI 0.53–0.88, *p* = .003). Similar but weaker associations were observed for the main findings in the secondary analyses.

**Conclusions:**

We found weak evidence to suggest that genes previously associated with TD are associated with sun exposure in children of European ancestry from southwest England.

**Electronic supplementary material:**

The online version of this article (10.1186/s12881-018-0575-z) contains supplementary material, which is available to authorized users.

## Background

The results of a UK study suggest that tanning makes people (particularly women) feel healthier and more attractive [1]. Tanning is the action of acquiring a darker skin color by exposing the skin to ultraviolet (UV) radiation. UV exposure (either by sun exposure or tanning equipment such as sun beds) can cause skin damage which is associated with the development of all major types of skin cancer [2–4]. Research findings suggest that the majority of people who tan, do so despite being aware of the associated health risks [5].

Continued tanning despite knowledge of health risks could be explained by dependence. In recent research, some tanners have met criteria for substance dependence defined by the Diagnostic and Statistical Manual (DSM) and the tanning-modified Cut Down, Annoyed, Guilt Eye-opener (mCAGE) Substance Abuse Screening Tool, suggesting that individuals can become tanning dependent [6–9]. Research also suggests that tanners can experience symptoms of dependence such as withdrawal [10]. In a sample of 400 participants from a university population in the USA, 27% were found to be tanning dependent [11] and in a study of frequent tanners, 26% met revised CAGE criteria and 53% met modified DSM-IV-TR criteria for tanning dependence (TD) [12]. Additionally, TD has been found to be associated with having an increased likelihood of being sun burnt in the last year and a lower likelihood of displaying protective behaviors such as covering the skin using clothing, avoiding being exposed to the sun, and using sun block/cream [11].

The well-established health risks of exposure to UV radiation combined with the relatively high prevalence of TD among tanners presents an issue for public health which needs resolving with targeted public health messages to prevent the development of TD without blaming the individual. A recent development in the genetic underpinnings of TD may allow researchers to identify pathways to dependency; Cartmel and colleagues found an association between TD and the patched domain containing 2 (*PTCHD2)* gene [13]. Additionally, several single nucleotide polymorphisms (SNPs) in the Ankyrin Repeat and Kinase Domain Containing 1 (*ANKK1*) and Dopamine Receptor D2 (*DRD2*) genes which are associated with substance dependence have also been found to be associated with ever tanning [14]. Sun exposure in childhood has previously been linked to genetic scores of pigmentation/tanning ability whereby having skin that burns instead of tanning is associated with limiting sun exposure by the use of sun block and covering the skin [15]. There may also be other genetic factors which influence childhood sun exposure besides carrying alleles which are associated with fairer skin; SNPs related to TD may influence exposure. By exploring the association between SNPs related to TD and sun exposure in children, we can determine whether there is a genetic susceptibility which may increase sun exposure in childhood. Displaying poorer protective behaviors against sun exposure in childhood could be a key indicator of the tendency to develop TD in later life as lower protective behaviors in adulthood are associated with TD [11].

However, children are not autonomous, their behavior and choices are normally limited by the actions and decisions of their parents. For this reason, exploring the association between mother’s SNPs and their child’s sun exposure could also be informative for public health messages to advise mothers who are at risk of being TD and whose children may be affected.

The aim of this research was to explore the association between SNPs related to TD (in both children and their mothers) and sun exposure in childhood.

## Methods

### Study population

The Avon Longitudinal Study of Parents and Children (ALSPAC) is a large, prospective cohort study which initially recruited 14,541 pregnant women whose expected delivery dates were from 1st April 1991 to 31st December 1992. Of the 14,062 live births, 13,988 children were alive at 1 year. This study has previously been described in detail [16, 17]. Additionally, the study website contains details of all the data that is available through a fully searchable data dictionary: http://www.bristol.ac.uk/alspac/researchers/access/.

Questionnaire responses from 7297 mothers were collected containing measures of their child’s sun exposure at age 8. The number of participants who answered each individual question and the sociodemographic data for the sample can be found in Table 1. The analysis was restricted to participants of European ancestry to reduce the confounding effects of darker skin tones and population stratification.

**Table 1:** Sex, age, sun exposure and pigmentation of the children and mothers in the study population

Variable (*N*)	%	*n*
Female (11,524)	48.5	5586
Had been badly sun burnt when aged 8 (7297)	1.7	124
Had been badly sun burnt between ages 0 and 8 (6600)	5.0	327
Number of days the child was in the sun for 4 or more hours at 8 years (6679)		
None	4.2	283
Less than 10	19.5	1303
10–19	23.3	1554
10–29	25.5	1702
30–39	15.1	1008
40 or more	12.4	829
Child normally wore a hat while out in the sun at 8 years (7206)		
Never	5.3	385
Sometimes	36.2	2606
Usually	39.2	2828
Always	19.3	1388
Child normally wore something to keep skin covered in the sun at 8 years (7193)		
Never	2.3	162
Sometimes	36.8	2648
Usually	44.5	3198
Always	16.5	1185
Child normally used sun cream whilst out in the sun at 8 years (7217)		
Never	0.9	64
Sometimes	11.2	810
Usually	32.6	2354
Always	55.3	3989
Factor of sun cream the child normally used at 8 years (7039)		
4–7	0.9	64
8–14	6.2	433
15–20	31.7	2232
21–25	16.4	1154
25+	44.8	3156
Star rating of sun cream child usually used at 8 years (1152)		
Low	29.3	337
High	70.8	815
Child usually avoided the midday sun at 8 years (7188)		
Never	8.1	580
Sometimes	36.1	2596
Usually	39.8	2861
Always	16.0	1151
Frequency of sun cream application at 8 years (7059)		
Once only	10.9	767
Every 3 to 4 h	43.1	3045
Every 2 h	34.9	2464
Every hour	10.1	711
Every half hour	1.0	72
Child’s skin pigmentation (7744)		
Always burns, never tans	0.9	72
Burns easily, rarely tans	9.6	743
Doesn’t change	4.0	312
Tans easily, rarely burns	40.0	3098
Always tans, never burns	17.3	1337
Can’t say [their] skin is always protected	28.2	2182
Mother’s eye color (3922)		
Blue	39.6	1553
Green	22.2	870
Brown	25.8	1011
Grey	5.8	227
Other	6.7	261
Means and SDs	Mean	SD
Age in months at questionnaire at 8 years (7297)	105	3

Sun exposure variables were reported by the children’s mothers

### Measures

The questionnaires used were created specifically for ALSPAC (Additional file 1).

Sex of the child was obtained from obstetric records. When the child was 6 years old, data was collected from the mother regarding the child’s skin pigmentation. Responses were categorized as: ‘always burns, never tans’; ‘burns easily, rarely tans’; ‘doesn’t change’; ‘tans easily, rarely burns’; ‘always tans, never burns’; and ‘can’t say [their] skin is always protected’.

Measures of sun related behaviors were collected via questionnaire at 8 years old. When the child was age 8, mothers were asked via questionnaire whether their child normally wore a hat, wore something to keep covered, used sun block or sun cream and whether they avoided the midday sun when out in the sun. Responses were categorized in the questionnaire as: ‘Yes, always’, ‘Yes, usually’, ‘Yes, sometimes’, and ‘No, never’. Data on the factor and star rating of the sun block or sun cream used were also gathered as well as the frequency of application. Factors of sun block or cream were categorized in the questionnaire as 1–3, 4–7, 8–14, 15–20, 21–25, and 25+. Categories 1–3 and 4–7 were combined in analyses due to limited responses. Star ratings of sun block ranged from one to four with four providing the greatest protection and one providing the least. Mothers also responded to questions about whether their child had been badly sun burnt when the child was 8 years old or ever up to the age of 8 (yes or no) and the number of days they had spent more than four hours in the sun during the whole year when they were 8 years old (responses were categorized in the questionnaire as: none, 1–9, 10–19, 20–29, 30–39, 40 or more). The age of the child in months at the time of questionnaire completion was also reported.

When the young person was 18 years old, the mother responded to a questionnaire regarding their own eye color (response options: blue, green, brown, grey, other).

### Genetic data

The genotyping methods have previously been described by Bonilla and colleagues [18]. Genome-wide genotypic data for the children were generated by Sample Logistics and Genotyping Facilities at the Wellcome Trust Sanger Institute (Cambridge, UK) and the Laboratory Corporation of America (Burlington, NC, USA) with support from 23andMe (Mountain View, CA, USA) using the Illumina HumanHap550 quad chip. Using the Illumina Human660W quad array, the mothers were genotyped at CNG (Centre National de Génotypage). Quality control consisted of excluding all individuals with ambiguous sex, of non-European ancestry, extreme heterozygosity, cryptic relatedness (IBD > 0.125 in mothers, IBD > 0.1 in children), high missingness (missingness > 5% in mothers, missingness > 3% in children) and insufficient sample replication (IBD < 0.8), and all SNPs with genotyping rate < 95%, MAF < 1%, or out of Hardy-Weinberg equilibrium (*p* < 1 × 10^− 6^ in mothers, *p* < 5 × 10^− 7^ in children). 477,482 SNP genotypes in common between the sample of mothers and sample of children were combined. Genotypic data was then phased with ShapeIT v2.r644, which utilizes relatedness during phasing, and was jointly imputed using IMPUTE v2.2.2 and phased haplotype data from the 1000 Genomes reference panel (phase 1, version 3, Dec 2013 release). Imputation was based on 465,740 SNPs and all 2186 reference haplotypes.

From the genome-wide data we selected 20 SNPs for analysis (listed in Additional file 2: Table S1) based on previous findings from Cartmel and colleagues [13] and Flores et al. [14]. These SNPs did not show strong evidence for association following correction for multiple comparisons in the original studies (which had small sample sizes) but the *p*-values were below 5 × 10^− 4^ or were below 0.05 and were also within or around substance dependence candidate genes.

One from each pair of SNPs which were in high linkage disequilibrium (r^2^ > .8, *n* = 2) were excluded from analyses. Data was unavailable for one SNP, leaving 17 SNPs out of the 20 originally selected to be included in the final analysis. The 17 SNPs were located in 13 different genes (see Table 2 and Additional file 2: Table S1). The SNPs used were well imputed (r^2^ > .8), in Hardy Weinberg equilibrium and did not substantially differ in allele frequency from the allele frequency in HapMap individuals of European descent (CEU).

**Table 2:** Number of associated sun exposure variables for children and mothers by gene before Bonferroni correction

	Number of sun exposure variables associated (*p* < .05) before Bonferroni correction							
Gene (No. SNPs tested)	Child ^a^	Child ^b^	Mother ^a^	Mother ^b^	Child ^c^	Child ^d^	Mother ^c^	Mother ^d^
ALDH1B1 (1)	1	1	1	1	1	0	2	1
ANKK1 (3)	1	1	0	1	1	2	1	1
DISP3 - PTCHD2 (1)	0	1	0	0	0	0	1	1
DRD2 (3)	1	1	3	3	2	1	2	4
HMHB1 (1)	0	1	0	1	1	0	2	1
KIAA1462 (1)	0	0	0	1	3	1	0	2
LY75 (1)	0	0	0	0	1	1	1	2
NCF4 (1)	1	0	1	0	1	1	0	0
OPRM1 (1)	3	1	1	2	0	0	1	0
SPATC1 (1)	2	2	0	1	2	0	0	0
TMC7 (1)	1	1	0	0	0	0	1	0
TMPRSS12 (1)	0	1	1	1	0	1	0	0
VAPA (1)	5	1	1	0	2	0	0	0
Total	15	11	8	11	14	7	11	12

^a^adjusted for age and principal components

^b^adjusted for age, principal components, and the mother’s/child’s genotypes

^c^adjusted for age, principal components, sex and pigmentation

^d^adjusted for age, principal components, sex, pigmentation and the mother’s/child’s genotypes

A total of 10 sun exposure variables were tested per SNP

### Ethics, consent and permissions

Ethical approval for the study was obtained from the ALSPAC Ethics and Law Committee and the Local Research Ethics Committees (http://www.bristol.ac.uk/alspac/researchers/research-ethics/). Written informed consent was obtained from the child’s mother on behalf of the mother and child.

### Statistical analyses

The associations between the chosen SNPs of both the children and their mothers (exposure) and sun exposure of the children (outcome) were assessed in a series of logistic regressions. Ordinal logistic regressions were used for analysis including more than two ordinal outcome categories. Analyses were adjusted for participant age in months at the time of the questionnaire completion and the top eight principal component variables that reflect population stratification.

Additionally, models were run including both the genotype of the child and the genotype of the mother in order to adjust for the effect of both. In these fully adjusted models, principal components (1–2) of both mother and child were adjusted for.

The above models were repeated to further adjust for sex and available pigmentation variables (eye color for mothers [skin pigmentation data not available] and skin pigmentation for children). These models were considered as secondary analysis to assess whether the findings supported the primary analysis as including these covariates dramatically reduced the sample size and therefore the power to detect associations.

Results were interpreted using Bonferroni adjustment where *p* < .05/17 = .003. The Bonferroni correction only takes account of the 17 different SNPs, the 10 sun exposures were not included in the correction as some were moderately correlated (correlation coefficients ranging from 0.01–0.57, most coefficients> 0.20, Additional file 2: Table S2) and so were not considered independent measures. Furthermore, as these are exploratory analyses rather than hypotheses driven, the focus of these results is not on *p*-value thresholds [19].

## Results

The children included in the analyses were 52% male and were aged 8 years on average (Table 1). Further demographic information reported by the mothers about the children’s sun exposure as well as information relating to the pigmentation of the children and their mothers can be found in Table 1.

Out of the 17 SNPs examined, 15 SNPs in 8 genes in the children (11 SNPs in 9 genes after adjusting for the mother’s genotype) and 8 SNPs in 6 genes in the mothers (11 SNPs in 8 genes after adjusting for the child’s genotype) showed evidence of association with at least one of the 10 children’s sun exposure variables before applying a Bonferroni correction for multiple comparisons (*p* < .05; Table 2 and Additional file 2: Table S3). Two out of three SNPs tested in the *ANKK1* gene (rs1003641 and rs7118900) and all three SNPs tested in the *DRD2* gene (rs12364283, rs12422191 and rs2440390) in children or mothers were associated with at least one of the sun exposure variables (Additional file 2: Table S3). Among the ALSPAC children, five sun exposure variables were associated with one SNP (rs29132) in the *VAPA* gene (2 after adjusting for sex and pigmentation) and three sun exposure variables were associated with one SNP (rs650662) in the Opioid Receptor Mu 1 (*OPRM1*) gene (0 after adjusting for sex and pigmentation, Table 2 and Additional file 2: Table S3). After adjusting for sex and pigmentation, one SNP in the *KIAA1462* gene (rs2887510) was associated with three sun exposure variables. For information regarding the number of SNPs associated with each sun exposure variable refer to Additional file 2: Table S4.

Following adjustment for participant age and the top eight principal component variables, and Bonferroni correction for multiple comparisons (*p* < .05/17 = .003), only one SNP in the children, rs60050811 found in the Spermatogenesis and Centriole Associated 1 *(SPATC1)* gene, showed evidence of being associated with sun exposure (whether the child usually used sun cream at 8 years old) (OR per C allele = 1.34, 95% CI 1.11–1.62, *p* = .0025). When adjusted for the mothers’ genotype the effect size was greater, however, the evidence for this association was weakened (Table 3).

**Table 3:** Association of tanning dependent related single nucleotide polymorphisms and sun exposure variables

SNP (RA)	Outcome	Partly adjusted				Fully adjusted			
		OR	95% CI	*p*	*n*	OR	95% CI	*p*	*n*
Childs SNPs									
rs60050811 (C)	Usually used sun block or cream when out in the sun at age 8 years	1.34	1.11–1.62	.0025	4983	1.43	1.09–1.88	.0106	3438
Mothers SNPs									
rs650662 (A)	Factor of sun block or cream the child usually used at age 8 years ^a^	0.89	0.82–0.96	.0020	4524	0.89	0.81–0.99	.0291	3459
rs2073478 (T)	Star system rating of child’s sun block or cream ^b^	0.68	0.53–0.88	.0028	741	0.68	0.49–0.94	.0181	585
Analyses adjusted for sex and pigmentation									
Childs SNPs									
rs60050811 (C)	Usually used sun block or cream when out in the sun at age 8 years	1.43	1.01–1.87	.0074	2549	1.29	0.90–1.86	.2651	1892
Mothers SNPs									
rs650662 (A)	Factor of sun block or cream the child usually used at age 8 years^a^	0.86	0.77–0.96	.0057	2385	0.88	0.77–1.01	.0627	1919
rs2073478 (T)	Star system rating of child’s sun block or cream ^b^	0.58	0.40–0.82	.0021	416	0.58	0.38–0.89	.0131	338

*RA* Risk allele (increases the risk of becoming TD, allele used based on findings from Cartmel et al. [13]); *SNP* Single nucleotide polymorphism

The partly adjusted model adjusted for the mothers’ or child’s principal components (1–8 in the mother’s and child’s analysis respectively), and the age of the child (in months) when the questionnaire was completed. The fully adjusted model was adjusted for both the child and mother’s SNPs and principal components (1–2) and the age of the child (in months) when the questionnaire was completed

^a^ 0 = factor 0–7; 1 = factor 8–14; 2 = factor 15–20; 3 = factor 21–25; 4 = 25+

^b^ 0 = star rating 1–3, 1 = star rating 4

One of the mothers’ SNPs (rs650662) in the *OPRM1* gene was associated with a reduced odds of their child using a higher factor of sun cream at 8 years old (OR per A allele = 0.89, 95% CI 0.82–0.96, *p* = .002) and one SNP in the Aldehyde Dehydrogenase 1 Family Member B1 (*ALDH1B1*) gene (rs2073478) was associated with a decreased odds of their child using a sun block or cream with the highest star system rating (4) compared to any other rating (1–3)(OR per T allele = 0.68, 95% CI 0.53–0.88, *p* = .003). However, it should be noted that there were much fewer responses to this question than others in the questionnaire and therefore only 741 participants were included in the partly adjusted model. When adjusted for the children’s genotype the evidence for these associations was weakened (Table 3), but the effect remained the same.

In the secondary analyses, where sex and pigmentation were included in the analyses, the findings were similar but weakened in terms of the effect size, strength of the effect or both (Table 3). However, the inclusion of these covariates resulted in a dramatic reduction in sample size (roughly 50%) and therefore power to detect an effect. Stratifying by sex rather than adjusting for sex indicated that the effects seen may be stronger in males but again, this further reduced the sample size and therefore the power (Additional file 2: Table S5).

To assess whether the season in which participants completed the questionnaire may have influenced responses, all the regression analyses were further adjusted for the month of questionnaire completion. There were no substantial differences between the results shown and the further adjusted results (results not shown).

## Discussion

The results provide weak evidence that SNPs in the *SPATC1* gene in children, and the *OPRM1* and *ALDH1B1* genes in their mothers are associated with the likelihood of sun exposure in childhood.

We found more associations than would be expected by chance at a 5% alpha level (i.e., one at most) between the children’s *VAPA* and *OPRM1* SNPs and multiple sun exposure variables in the children. However, it should be noted that these behaviors are likely to be highly correlated and therefore a higher number of associations is more likely than if the behaviors were completely independent of each other. After adjustment for sex and pigmentation, there were no longer more associations than we would expect by chance. Although, this could be due to the reduction in sample size and therefore power to detect an association. SNPs in the genes *ANKK1*, *DRD2* and *PTCHD2*, which were reported previously as potentially underlying TD [13, 14], did not show an association with sun exposure variables beyond what would be expected by chance, nor were they associated with the sun exposure outcomes after Bonferroni correction.

Following Bonferroni correction, a SNP (rs60050811) in the *SPATC1* in the children was associated with use of sun block or cream when out in the sun at 8 years old. In the study by Cartmel et al. [13] the C allele was associated with TD (OR for each additional C allele adjusted for age and sex = 4.13), in ALSPAC the C allele was associated with an increase in the odds of using sun cream when in the sun. A potential explanation for this difference could be that these children may be less likely to adhere to other sun protective behaviors such as keeping covered with clothing and parents may mitigate this by ensuring their child is protected with sun cream. The rs650662 SNP in the *OPRM1* gene in the mothers was associated with the factor of sun cream children normally used at 8 years. *OPRM1* is associated with substance dependence [20] which supports the notion that this gene is related to TD. The gene has also been shown to be associated with skin pigmentation [21] which could explain the association as some skin types are intolerant of tanning. However, we found no clear evidence of an association between rs650662 (or rs6005081/rs2073478) and pigmentation in our study (Additional file 2: Table S6). The risk allele (A) was associated with an 11% decrease in the odds of using a higher, more protective, factor of sun cream. Interestingly, Cartmel et al. [13] found that there was an increased risk of being TD with each A allele at this SNP (OR for each additional A allele adjusted for sex and age = 1.64). Similarly, the rs2073478 SNP in the ALDH1B1 gene was associated with a 32% decrease in the odds of the sun cream being used by mothers on their child having the highest and most protective star rating (4 star compared to 1–3). Again, the risk allele (T) was associated with increased risk of being TD (OR for each additional T allele adjusted for sex and age = 2.22) in Cartmel and colleagues’ findings [13]. This suggests that mothers with an increased risk of TD are using lower factors of sun cream with lower star ratings on their children which could be because they are using lower factor sun creams with lower star ratings themselves in order to enhance their tan. This is consistent with the hypothesis that mothers with TD may protect their child less from UV damage than mothers without TD. When adjusting for the mother’s and children’s genotypes, respectively, the evidence for all three of the above findings was weakened.

In our secondary analyses adjusting for sex and pigmentation, the results were similar but weakened. This could indicate that sex and pigmentation could be modifying the effect of the TD genotype on sun exposure which would be supported by previous evidence showing that sex and pigmentation affect indoor tanning [22]. However, the dramatic reduction in sample size that accompanied the inclusion of these covariates in the model resulted in a reduction in the power to detect an effect.

Having access to the mother’s data for this study allowed us to explore the influence a mother’s genotype may have on their child’s sun exposure as well as the effect of the child’s genotype. Interestingly the size of the effects seen in the children changed after adjustment for the maternal genotype (although the strength of evidence was weakened), whereas the effect of the mother’s genotype was not modified after accounting for the child’s genotype, suggesting that the mother’s genetic background may play a role in the children’s exposure to the sun. Adjusting for the mothers’ and children’s SNPs in these analyses provides an insight into the possible effects one may have on the other in terms of the child’s behavior, but it is important to note that there is a risk of introducing collider bias when performing this adjustment [23], so the results should be interpreted with caution.

The results of this study should be carefully considered/evaluated due to the weak evidence of associations found between polymorphisms in potential TD genes and tanning related behavior in childhood. The study by Cartmel and colleagues reported a strong association of TD with the *PTCHD2* gene in a gene burden test, but no SNPs showed clear evidence of association following correction for multiple comparisons [13]. Flores and colleagues on the other hand, showed associations between ever indoor tanning and substance dependence related SNPs in analyses without correction for multiple comparisons but we found no clear associations between these SNPs and the children’s sun exposure [14]. This difference in findings could be due to the focus on childhood behavior in this study rather than behavior in adulthood when addictive behavior is more likely to have been established. Alternatively, the outcome of interest (ever used indoor tanning) in the study by Flores et al. [14] may have not been a good measure of tanning dependence and neither study have validated their results nor demonstrated evidence of an association after correction for multiple comparisons.

It should be noted, that this study used data from a large longitudinal data set (with a much larger sample size than the sample sizes of previous studies in the area) and therefore we had greater power to detect an effect which may explain why there are differences between our findings and those of previous reports. However, the use of maternal reports of sun-exposure may reduce the validity of the data as mothers may not be aware of their child’s exposure at all times such as when they are at school. Although it could also be argued that children spend the majority of their time inside during the school day, mothers can protect their child from sun exposure during the day by applying sun cream before school, and mothers may observe sun burn when their child returns from school. At present, there is no data available on TD or indoor tanning in ALSPAC participants which prevents the direct comparison of these findings and previous findings in the area, but the longitudinal nature of the ALSPAC study provides the opportunity for future data collection to assess whether these at risk children have developed TD later in life. The cohort are now 26 years old, meaning that if we can acquire this data we could more accurately assess their TD without their mothers’ mediating effects and assess the association between their genotypes and TD as well as the association between their behaviors in childhood and TD.

## Conclusions

The weak findings in this study provide little support for a genetic predisposition to TD affecting sun exposure in childhood. Similarly, there was limited evidence of an association between mothers’ TD genotype and their child’s sun exposure. If future evidence supports these findings, it may provide reassurance to public health officials concerned about maternal influence on children’s sun exposure and lead to a reframing of public health messages. Further research could explore the effect of paternal genotype on childhood behavior and whether this could mediate any of the effects found.

## Additional files

Additional file 1: Questionnaire of sun exposure completed by mothers about their children at 8 years old. This file contains the questionnaire used to assess children’s sun exposure at 8 years. The questionnaire was completed by the mothers about their children. (DOCX 188 kb)

Additional file 2: Supplementary data for the influence of maternal and own genotype at tanning dependence-related SNPs on sun exposure in childhood. This file contains **Tables S1–S6** which includes a list of SNPs and relevant details) selected for analysis, a correlation matrix of sun exposure variables, a complete table of the associations between tanning dependence related SNPS and sun exposure, and the number of SNPs associated with each measured sun exposure variable prior to Bonferroni correction. Additionally, the file contains a table of associations between SNPs and pigmentation variables, and a table of associations between tanning dependence related SNPS and sun exposure stratified by sex. (XLSX 331 kb)

## Abbreviations

ALSPAC: Avon longitudinal study of parents and children
*ANKK1*: Ankyrin repeat and kinase domain containing 1
CNG: Centre National de Génotypage
*DRD2*: Dopamine receptor D2
DSM: Diagnostic and statistical manual
GWAS: Genome wide association study
IBD: Identity-by-descent
MAF: Minor allele frequency
mCAGE: The tanning-modified cut down, annoyed, guilt eye-opener substance Abuse Screening tool
*OPRM1*: Opioid receptor Mu 1
*PTCHD2*: Patched domain containing 2
SNP: Single nucleotide polymorphism
*SPATC1*: Spermatogenesis and centriole associated 1
TD: Tanning dependence
UV: Ultraviolet
