# Supplementary figures and images for: Influence of maternal and own genotype at tanning dependence-related SNPs on sun exposure in childhood
Source: BMC Med Genet. 2018 Apr 12;19:62. doi: 10.1186/s12881-018-0575-z (PMC5898059; doi:10.1186/s12881-018-0575-z)

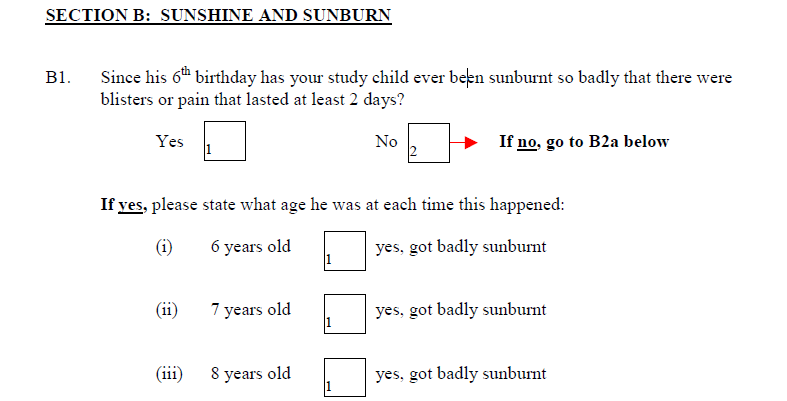


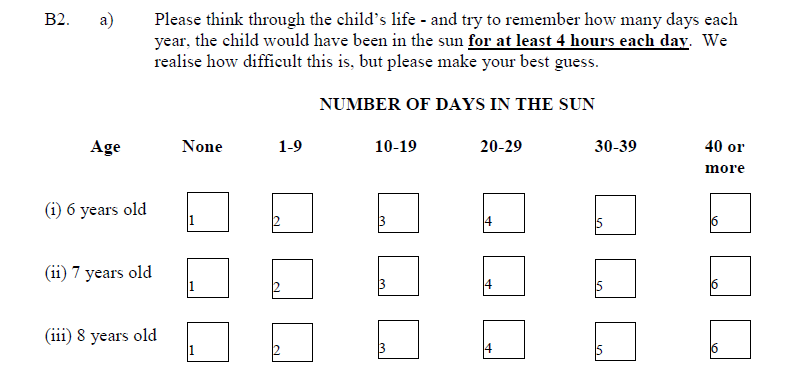


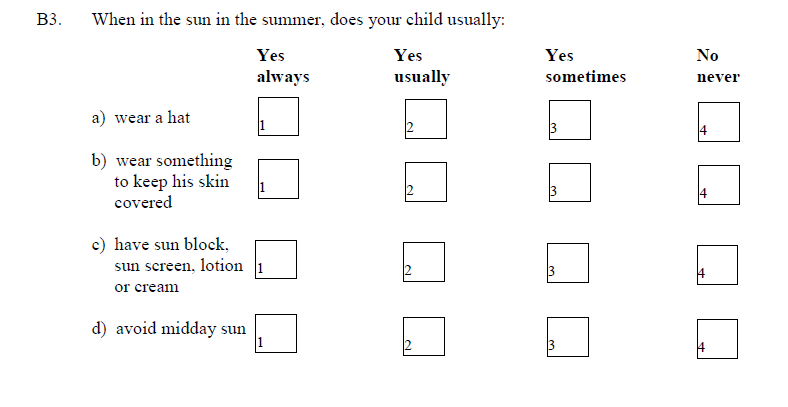


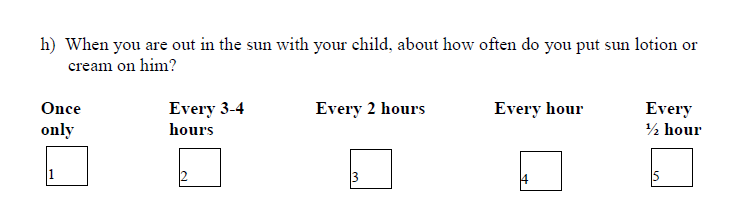

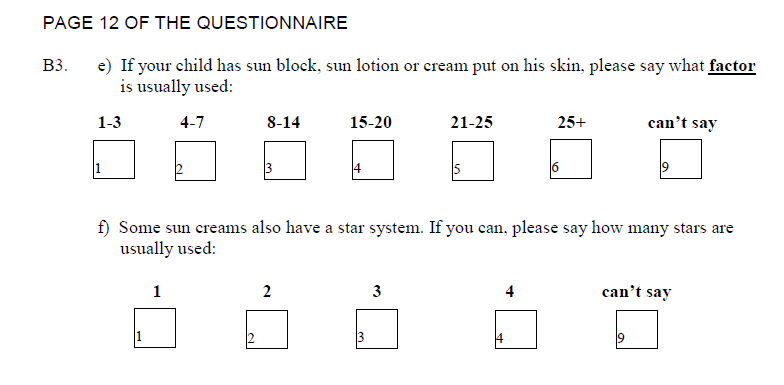

Supplement: Supplementary file 1 — Questionnaire of sun exposure completed by mothers about their children at 8 years old. This file contains the questionnaire used to assess children’s sun exposure at 8 years. The questionnaire was completed by the mothers about their children. (DOCX 188 kb) [file 12881_2018_575_MOESM1_ESM.docx]
